# Swimming ability of cyprinid species (subfamily schizothoracinae) at high altitude

**DOI:** 10.3389/fphys.2023.1152697

**Published:** 2023-07-20

**Authors:** Lu Cai, Yingping Huang, David Johnson, Minne Li, Rui Liu, Wangbin Hu, Yao Jin, Xiaojuan Chen, Jiangping Tao, Xuan Zou, Yiqun Hou

**Affiliations:** ^1^ Key Laboratory of Ecological Impacts of Hydraulic-Projects and Restoration of Aquatic Ecosystem, Institute of Hydroecology, Ministry of Water Resources and Chinese Academy of Sciences, Wuhan, China; ^2^ Engineering Research Center of Eco-Environment in Three Gorges Reservoir Region, China Three Gorges University, Yichang, China; ^3^ School of Natural Sciences and Mathematics, Ferrum College, Ferrum, VA, United States; ^4^ Northwest Engineering Corporation Limited of PowerChina, Xian, China; ^5^ GNSS Research Center, Wuhan University, Wuhan, China

**Keywords:** swimming ability, schizothoracinae, altitude, hypoxia, critical swimming speed, burst speed

## Abstract

The primary objective of this investigation was to study the effect of altitude on fish swimming ability. Different species were tested to ensure that the differences observed are not associated with a single species. Fish critical swimming speed and burst speed were determined using stepped-velocity tests in a Brett-type swimming respirometer. Based on the effects of water temperature and dissolved oxygen, it is clear that the swimming ability of fish decreases as altitude increases. Further, because the effects of high altitude on fish physiology go beyond the effects of lower temperature and dissolved oxygen, we recommend that fish swimming ability be tested at an altitude similar to the target fishway site to ensure the validity of fish data used for fishway design.

## Introduction

Numerous fish species of the Schizothoracinae family (subfamily of Cyprinidae) are endemic to Asian plateaus, including the Qinghai-Tibet Plateau and its environs (Hou et al., 2018; Li et al., 2021). This region is characterized by high mountains and large rivers, and the terrain is steep and highly variable. The average altitude (∼4000 m) in the Qinghai-Tibet Plateau is far higher than surrounding regions of the same latitude. Differences in altitude result in differences in air temperature and atmospheric pressure that lead to differences in river water temperature and dissolved oxygen (DO) level. The differences in river conditions, in turn, have important effects on fish physiology (Davis et al., 1963; Ojanguren and Brana, 2000; Penghan et al., 2014), including swimming ability and behavior.

A number of studies have reported that hypoxia decreases fish swimming ability (Davis et al., 1963; Farrell et al., 1998; Penghan et al., 2014). GardunoPaz et al. (2020) reported that, based on the analysis of metabolic rate and hypoxia tolerance compared to other species at the same temperature, Girardinichthys multiradiatus is well adapted for the hypoxia associated with their high-altitude habitat. But overall, reports on hypobaric hypoxia at high altitudes are rare in the fish literature. While little is known about the effects of hypobaric hypoxia on fish, the effects on humans and animals have been well documented. If nutritional status is adequate, metabolic rate is limited by oxygen and athletic performance decreases as hypobaric hypoxia becomes more severe. Among those who visit high altitudes, a decline in functional capacity is a near universal experience (Peronnet et al., 1991; Bartsch and Gibbs, 2007). Hypobaric hypoxia has also been shown to result in nerve synapse dysfunction and neurodegeneration in humans (Kushwah et al., 2018). If these physiological effects occur in fish, swimming ability would be reduced. However, maintaining metabolic energy is the primary challenge posed to fish by hypoxic conditions, as 95% of the oxygen consumed by fish is used for adenosine triphosphate production (Richards, 2009). The effect of hypobaria on the swimming ability of fish inhabiting high-altitude rivers is not known, but adaptation to hypobaric conditions may occur. For example, to adapt to the Qinghai-Tibet plateau environment, Schizothoracinae species exhibit evolutionary changes such as scale degeneration, increased mucous gland secretion, and thickening of the subcutaneous adipose layer to facilitate swimming, predation, and cold resistance (Chang et al., 2010; Zhang et al., 2018; Ma et al., 2023).

Data on fish swimming ability has accumulated globally (Castro-Santos, 2005; Bunt et al., 2016; Sanz-Ronda et al., 2016; Katopodis et al., 2019), and ichthyologists and engineers use this data when setting criteria for fishway water velocities. Tests of swimming ability for this purpose generally includes critical swimming speed (U_crit_) and burst speed (U_burst_) (Beamish, 1978; Hammer, 1995; Plaut, 2001; Cai et al., 2018). The U_crit_ reflects the prolonged swimming ability (Hammer, 1995) and is used to set the average water velocity and fishway length. The U_burst_ reflects the maximum swimming speed, achievable for only brief periods (<20 s), is used to set the maximum water velocity of the fishway (Cai et al., 2018). Most of the data on swimming ability (especially U_crit_) were obtained in a respirometer with laminar flow. Although this can be problematic when applied to fishways with turbulent flow and heterogeneous hydraulic conditions (low-velocities zones, roughness elements such as boulders or baffles inducing turbulence, etc.), the respirometer can be used reliably to compare fish swimming ability under different conditions (Hammer, 1995).

Fish swimming ability varies greatly among species. Species in the same family may have similar swimming ability, given similar body size and shape, but ability also varies among species within families, e.g., within Salmonidae, Cyprinidae, and Acipenseridae (Verhille et al., 2014; Cano-Barbacil et al., 2020). Some species, or similar species of the same family or subfamily (e.g., Schizothoracinae, subfamily of Cyprinidae), are distributed at both high and low altitudes. Because air temperature and atmospheric pressure decrease with altitude, the results of fish swimming tests conducted at low altitude may not be appropriate for setting the design criteria of high-altitude fishways. Thus, it is important to know how altitude affects fish swimming ability.

The primary objective of this investigation was to study the effect of altitude on fish swimming ability using a fish respirometer. Different species were tested to ensure that the differences observed are not associated with a single species. The results supplement the basic scientific data on fish swimming behavior and provide a reference for fishway design for these species at high altitudes.

## Materials and methods

### Fish

Fish cages (22 m × 0.4 m × 0.3 m, mesh 4 mm × 4 mm) were used to catch fish for testing. The fish were transferred to cylindrical pools (0.8 m in diameter and 0.9 m deep, 10 fish per pool), approximately a kilometer from shore, and allowed to acclimate to conditions for a week prior to testing. Schizothoracinae species tested included *Schizothorax prenanti*, *Schizothorax chongi*, *Schizothorax nukiangensis*, *Schizopygopsis thermalis* and *Ptychobarbus kaznakovi*. The water used in the holding pools and respirometers was river water that was aerated after particles had been allowed to settle. Water changes were made twice a day to maintain the water temperature. The water temperature ranged from 12.9°C to 22.5°C (Table 1). Fish were fed daily with Tubificidae. The DO was measured with a DO analyzer (Hach HQ30d, Loveland, United States), and the DO was maintained at >70% saturation using an air pump. It should be noted that, while the relative DO level was maintained at >70% saturation at all altitudes, the absolute DO concentration decreases with altitude in direct proportion to the decrease in atmospheric pressure (Henry’s Law).

**TABLE 1:** Swimming test conditions and results (n = 10 for U_crit_ and U_burst_ tests).

Altitude (m)	Species	Temperature (^o^C)	Dissolved oxygen (mg/L)	Body length (m)	U_crit_ (bl/s)	U_burst_ (bl/s)
1326	*Schizothorax prenanti*	17.7–22.4	7.37–8.73	0.20 ± 0.07	5.10 ± 1.67	—
				0.29 ± 0.02	—	4.23 ± 0.57
1326	*Schizothorax chongi*	17.6–22.5	7.23–8.56	0.18 ± 0.04	5.92 ± 1.45	—
				0.14 ± 0.02	—	9.15 ± 1.59
1970	Schizothorax yunnanensis*	19.1–22.7	6.36–8.56	0.19 ± 0.03	6.13 ± 1.10	—
				0.19 ± 0.04	—	9.59 ± 2.36
1970	Schizothorax griseus*	11.5–13.5	8.45–9.02	0.19 ± 0.03	4.61 ± 1.11	—
				0.19 ± 0.03	—	6.36 ± 1.12
1970	Schizothorax lantsangensis*	12.2–13.4	8.48–8.95	0.23 ± 0.05	4.07 ± 0.80	—
				0.17 ± 0.02	—	6.25 ± 1.44
1970	Schizothorax lissolabiatus*	11.1–14.0	7.57–8.84	0.25 ± 0.06	3.74 ± 0.94	—
				0.27 ± 0.06	—	5.84 ± 1.97
3923	*Schizothorax nukiangensis*	12.9–14.7	4.38–5.99	0.22 ± 0.04	4.11 ± 0.67	—
				0.22 ± 0.06	—	4.81 ± 0.92
3923	*Schizopygopsis thermalis*	13.3–14.7	4.35–5.95	0.22 ± 0.03	3.77 ± 0.75	—
				0.21 ± 0.02	—	4.73 ± 0.80
3923	*Ptychobarbus kaznakovi*	12.9–14.2	4.50–5.96	0.22 ± 0.03	3.66 ± 0.52	—
				0.22 ± 0.04	—	4.75 ± 0.40

*Data from Cai et al., 2018.

The fish for this study were collected and tested at altitude during their spawning migration (April to July). This is the period when fish are active and more motivated to swim, and also the main operating season for fish passage facilities. Fish were tested at two altitudes in this study, 1326 and 3923 m, using a different Schizothoracinae species at each altitude (Table 1). Additionally, the data set from a previous study, conducted on four species at 1970 m using the same experimental protocol during fish spawning migration (April to June) (Cai et al., 2018) was included for comparison. Thus, this study analyzes the swimming ability of fish at three altitudes, 1326, 1970, and 3923 m.

#### Apparatus

The experimental apparatus was a Brett-type swimming respirometer. The total volume of the apparatus is 95 L with a rectangular 28 L swim chamber (0.7 m × 0.2 m × 0.2 m) (Cai et al., 2020). The entrance of the swim chamber is fitted with a multi-aperture (1 cm × 1 cm) flow rectifier (Lucite) that helps maintain laminar flow, and a wire grid at the back that prevents fish from exiting the chamber. The water velocity in the swim chamber, controlled with a propeller driven by a variable speed motor, was measured with a propeller flow velocity meter (LGY-II, Nanjing, China). Water used for the swimming tests was the same as in the holding pools. Peake and Farrell (2006) recommended a swim chamber length at least 2.1 times the test fish body length, in order to avoid constraining fish movement. In this study, the length of the swim chamber was 0.7 m, which ranges from 2.1 to 7.4 times the body length of the tested fish.

#### Fish swimming test

The stepped velocity tests were conducted at a water temperature and DO level similar to the test site river to reflect the ambient air temperature and atmospheric pressure at altitude. Stepped velocity tests were carried out to determine the U_crit_ and U_burst_ of each species. Fish were tested one at a time and each fish was only tested once. Fish were tested until 10 fish per test were swimming normally (as 0–2 fish in each group sometimes refused to swim). Before being tested, the fish were fasted for 2 days. After measuring body length, the fish was placed in the swim chamber and the water velocity was adjusted to 0.5 bl/s for a 2 h acclimation period (Cai et al., 2018). To begin the test, the water velocity was adjusted to the initial value of 1 bl/s and then increased by increments of 1 bl/s, at 15 min intervals for U_crit_ testing, and at 20 s intervals for U_burst_ testing (Brett, 1964; Cai et al., 2018). When a test fish stopped swimming, the water velocity was rapidly decreased to approximately 1 bl/s and the fish was encouraged to continue swimming by tapping the swim chamber. Among the different groups, the proportion of test fish requiring encouragement was 0%–30%, and they were encouraged once or twice. If the fish began swimming again within 5–10 s, the water velocity was increased rapidly back to test velocity and the test continued. If not, the fish was considered fatigued, and the test was over. The U_crit_ and U_burst_ were calculated based on the equation from Brett (1964) below:

$$U_{crit}=U_{p}+\left(t_{f}{/t}_{i}\right)\times U_{t}$$ (Equation 1)

Where U_p_ (bl/s) is the highest velocity at which fish swam for the entire time interval, U_t_ (bl/s) the incremental speed step, t_f_ (min) the time a fish swam at the fatigue velocity (i.e., last velocity step) and t_i_ (min) the prescribed time step.

### Data analysis

Results are reported as the mean value ± SD (standard deviation). Origin 9.0 software (OriginLab Corporation, United States) was used for data analysis. The level of significance was set at *p* < 0.05.

The variances of fish body length, U_crit_ and U_burst_ deviated from normality (*p* < 0.05) and then log was applied. The log transformed data were normal and homogenous (*p* > 0.05) and then the ANCOVA with altitude and length as covariate was conducted (The groups tested at each altitude included more than one fish species to help ensure that the observed differences in swimming ability were due to differences in altitude rather than differences between species).

U_crit_ and U_burst_ (bl/s) were treated as dependent variables. Independent variables included water temperature, DO, fish body length, as well as the interactions between altitude and water temperature, altitude and DO, water temperature and DO. Principal component analysis (PCA) was used to determine the relative importance of each environmental parameter (altitude, water temperature, DO) and each interaction pair (altitude × DO, altitude × temperature and DO × temperature) on swimming ability (U_crit_ and U_burst_). Among the principal components, PC1 and PC2 explained approximately 75% of the variance in the original variables. Thus, because they captured most of the information, we used only PC1 and PC2. The Pearson test was used to measure the correlation between environmental parameters and swimming ability, in which the closer the correlation coefficient is to 1, the more correlated are the variables. Principal component analysis and Pearson correlation analysis were conducted to better understand which altitude parameter (temperature, DO) most affected swimming ability.

## Results

**FIGURE 1:**
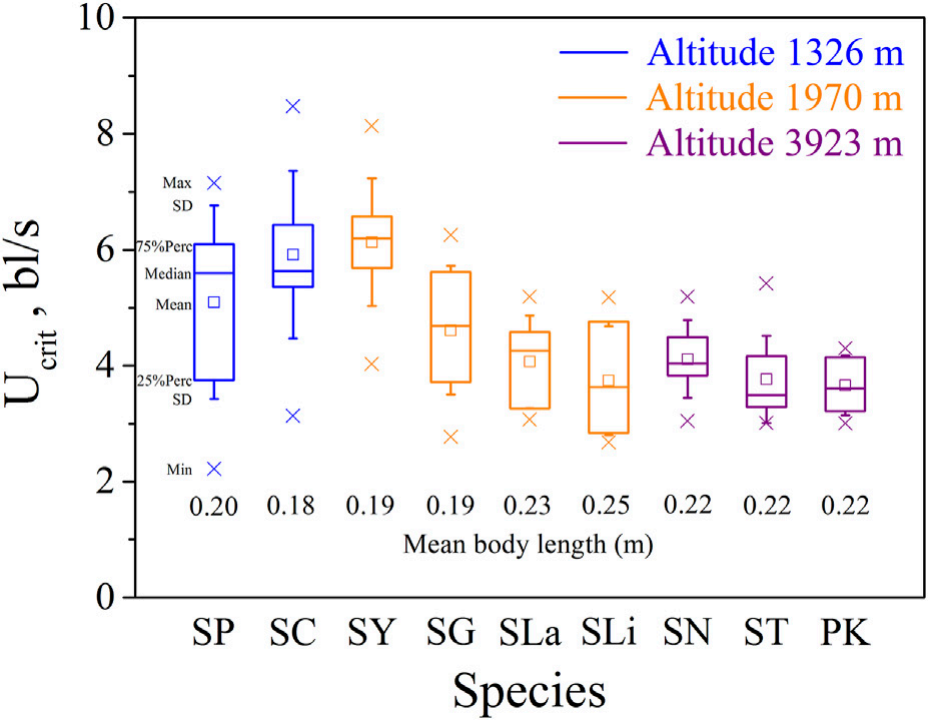
Maximum, Minimum, mean, median, quartile, and standard deviation of critical swimming speed U_crit_ for the nine species tested (n = 10). Species key: SP, *Schizothorax prenanti*; SC, *Schizothorax chongi*; SY, *Schizothorax yunnanensis*; SG, *Schizothorax griseus*; SLa, *Schizothorax lantsangensis*; SLi, *Schizothorax lissolabiatus*; SN, *Schizothorax nukiangensis*; ST, *Schizopygopsis thermalis*; PK, *Ptychobarbus kaznakovi*.

**FIGURE 2:**
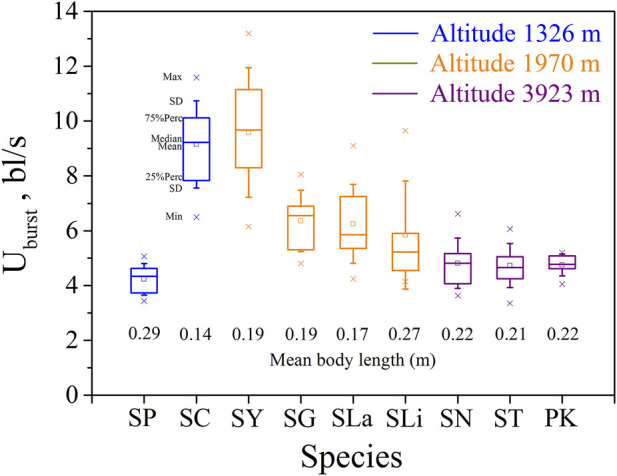
Maximum, Minimum, mean, median, quartile, and standard deviation of burst speed U_burst_ for the nine species tested (n = 10). Species key: SP, *Schizothorax prenanti*; SC, *Schizothorax chongi*; SY, *Schizothorax yunnanensis*; SG, *Schizothorax griseus*; SLa, *Schizothorax lantsangensis*; SLi, *Schizothorax lissolabiatus*; SN, *Schizothorax nukiangensis*; ST, *Schizopygopsis thermalis*; PK, *Ptychobarbus kaznakovi*.

**FIGURE 3:**
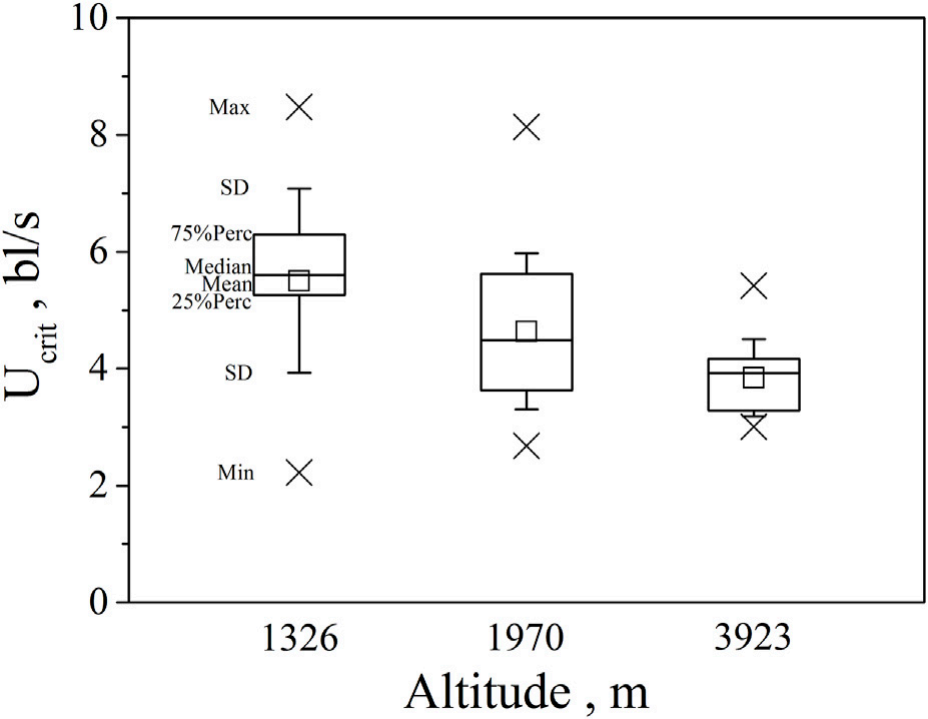
Maximum, Minimum, mean, median, quartile, and standard deviation of critical swimming speed U_crit_ for the three altitude group (n = 20, 40, 30 for altitude 1326, 1970, 3923).

**FIGURE 4:**
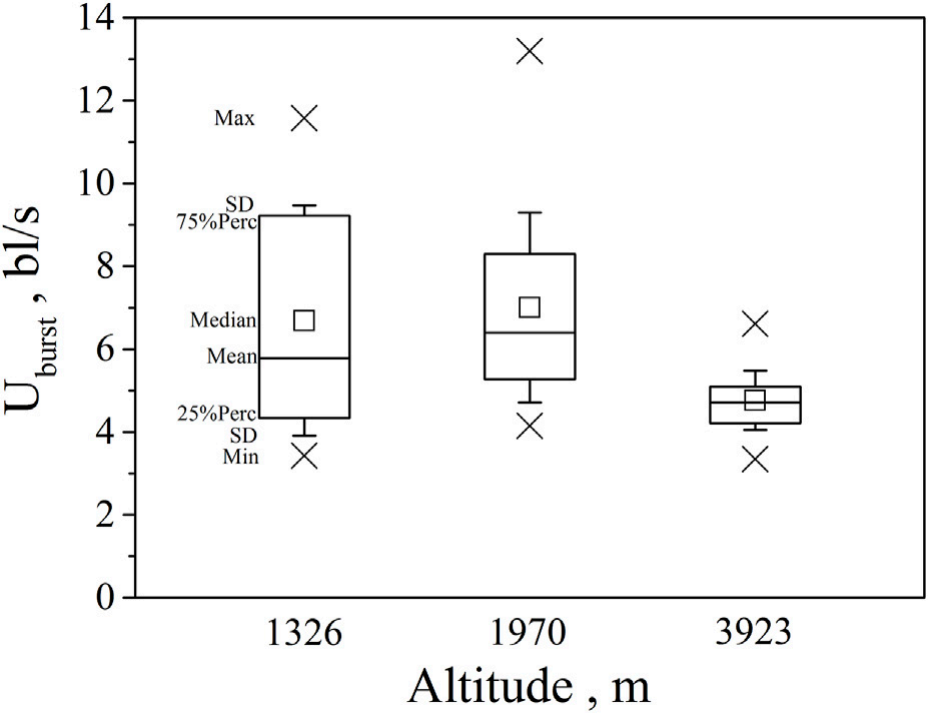
Maximum, Minimum, mean, median, quartile, and standard deviation of burst speed U_burst_ for the three altitude group (n = 20, 40, 30 for altitude 1326, 1970, 3923).

Swim test conditions and results are summarized in Tables 1, 2. The U_crit_ ranged from 2.22 bl/s to 8.48 bl/s, and U_burst_ ranged from 3.35 bl/s to 13.20 bl/s. The distributions of U_crit_ and U_burst_ data in each species are shown in Figures 1, 2, and the data distributions of each altitude group are shown in Figures 3, 4. We conducted a covariate analysis with altitude as the independent variable, U_crit_ as the dependent variable, and body length as the covariate. The results in Table 3 show that altitude significantly influences fish swimming ability (*p* = 0.005), while body length does not (*p* = 0.659). Furthermore, the mean values indicate that higher altitudes are associated with weaker swimming ability (Table 4). A covariate analysis was also conducted with altitude as the independent variable, burst swimming ability as the dependent variable, and fish body length as the covariate. The results (Table 5) again show that altitude significantly influences fish swimming ability (*p* = 0.000) and body length does not (*p* = 0.060), while the mean values again indicate that higher altitudes are associated with weaker swimming ability (Table 6). In addition, principal component analysis showed that body length explained only 7.71% and 8.57% of the variation in U_crit_ and U_burst_.

**TABLE 2:** Differences among altitude groups.

Altitude (m)	Temperature (^o^C)	Dissolved oxygen (mg/L)	Body length (m)	U_crit_ (bl/s)	U_burst_ (bl/s)	Source
1326	19.9 ± 1.5	8.00 ± 0.37	0.203 ± 0.067	5.51 ± 1.58	6.69 ± 2.78	This study
1970	14.6 ± 3.4	8.67 ± 0.60	0.209 ± 0.052	4.64 ± 1.33	7.01 ± 2.29	Cai et al. (2018)
3923	13.5 ± 0.4	5.24 ± 0.44	0.218 ± 0.038	3.85 ± 0.66	4.76 ± 0.71	This study

The altitude 1326 m group contains 2 species (Schizothorax prenanti and Schizothorax chongi), the 1970 m group contains 4 species (Schizothorax yunnanensis, Schizothorax griseus, Schizothorax lantsangensis, Schizothorax lissolabiatus), the 3923 m group contains 3 species (Schizothorax nukiangensis, Schizopygopsis thermalis, Ptychobarbus kaznakovi) in Schizothoracinae (a subfamily of Cyprinidae). Each fish was tested only once (U_crit_ test or U_burst_ test). The sample size for each test (U_crit_ test or U_burst_ test) was 10 individuals per species.

**TABLE 3:** Results of covariance analysis of log U_crit_ (m/s).

Source of difference	Sum of squares	*df*	Mean square	*F*	*p*
Intercept	0.016	1	0.016	2.696	0.104
Altitude	0.068	2	0.034	5.680	0.005
Body length	0.001	1	0.001	0.196	0.659
Residual	0.513	86	0.006		

*R* ^2^ = 0.117; Altitude is independent variable and body length is covariance.

**TABLE 4:** Average value of U_crit_ (m/s) under different altitudes.

Altitude	Average	Standard deviation	n
1326	0.97	0.17	20
1970	0.94	0.18	40
3923	0.83	0.10	30

**TABLE 5:** Results of covariance analysis of log U_burst_ (m/s).

Source of difference	Sum of squares	*df*	Mean square	*F*	*p*
Intercept	0.000	1	0.000	0.034	0.854
Altitude	0.278	2	0.139	19.139	0.000
Body length	0.026	1	0.026	3.636	0.060
Residual	0.625	86	0.007		

*R* ^2^ = 0.236; Altitude is independent variable and Body length is covariance.

**TABLE 6:** Average value of U_burst_ (m/s) under different altitudes.

Altitude	Average	Standard deviation	n
1326	1.36	0.25	20
1970	1.27	0.30	40
3923	1.01	0.15	30

Our major finding was that, although there was some overlap between species (Figures 1, 2) and between altitudes (Figures 3, 4), the U_crit_ and U_burst_ were significantly affected by altitude (*p* = 0.005 and *p* = 0.000 in Tables 3, 5), with the following specifics for each test. The U_crit_ of the low altitude group (5.51 ± 1.58 bl/s) was higher than those of the medium altitude group (4.64 ± 1.33 bl/s) and the high altitude group (3.85 ± 0.66 bl/s), and the U_crit_ of the medium altitude group was significantly higher than that of the high altitude group. The SD values for the U_burst_ of low and medium altitude groups were large, indicating high variability within the groups (Figure 4; Table 2). While the U_burst_ of the high altitude group (4.76 ± 0.71 bl/s) was lower than that of the medium altitude group (7.01 ± 2.29 bl/s), differences were not significant between the low altitude groups (6.69 ± 2.78 bl/s) and medium altitude groups.

**FIGURE 5:**
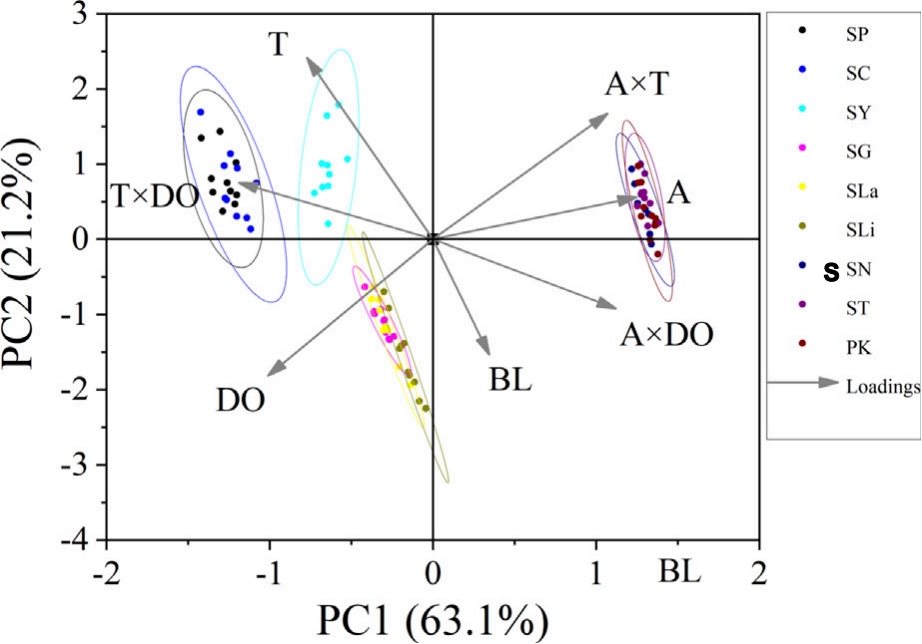
Principal component analysis for critical swimming speed U_crit_ (n = 10 for each species). PC1, principal component for X axis. PC2, principal component for Y axis. Factors: BL, body length; A, altitude; T, temperature; DO, dissolved oxygen. Species key: SP, *Schizothorax prenanti*; SC, *Schizothorax chongi*; SY, *Schizothorax yunnanensis*; SG, *Schizothorax griseus*; SLa, *Schizothorax lantsangensis*; SLi, *Schizothorax lissolabiatus*; SN, *Schizothorax nukiangensis*; ST, *Schizopygopsis thermalis*; PK, *Ptychobarbus kaznakovi*.

**FIGURE 6:**
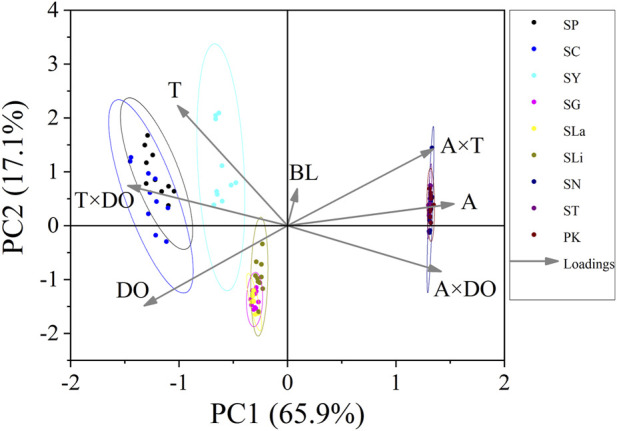
Principal component analysis for critical swimming speed U_burst_ (n = 10 for each species). PC1, principal component for X axis. PC2, principal component for Y axis. Factors: BL, body length; A, altitude; T, temperature; DO, dissolved oxygen. Species key: SP, *Schizothorax prenanti*; SC, *Schizothorax chongi*; SY, *Schizothorax yunnanensis*; SG, *Schizothorax griseus*; SLa, *Schizothorax lantsangensis*; SLi, *Schizothorax lissolabiatus*; SN, *Schizothorax nukiangensis*; ST, *Schizopygopsis thermalis*; PK, *Ptychobarbus kaznakovi*.

The Pearson test was used to determine the correlation between swimming speed (U_crit_ and U_burst_, bl/s) and each of the independent variables. The correlation coefficient of each factor is given in Table 7. The U_crit_ (bl/s) correlated negatively (correlation coefficient < 0) with respect to altitude, body length, altitude × temperature, altitude × DO, and temperature × DO, and positively (correlation coefficient > 0) with respect to temperature and DO. The U_burst_ (bl/s) correlated negatively (correlation coefficient < 0) with respect to altitude, body length, altitude × temperature, and altitude × DO, and positively (correlation coefficient > 0) with respect to temperature, DO, and temperature × DO. Specifically, U_crit_ (bl/s) increases as temperature and DO increase, and U_crit_ (bl/s) decreases as body length, altitude, altitude × temperature, altitude × DO and temperature × DO increase. U_burst_ (bl/s) increases as temperature, DO and temperature × DO increase, and U_burst_ (bl/s) decreases as body length, altitude, altitude × temperature, and altitude × DO increase. Principal component analysis indicated that each of these factors contributed to the variation in U_crit_ and U_burst_ (Table 8; Figures 5, 6). Except for body length, the variables had similar effects on swimming ability and their interactions also affected swimming ability.

**TABLE 7:** Pearson correlation analysis (all *p* < 0.05).

Factors	U_crit_ (bl/s)	U_burst_ (bl/s)
Body length	−0.742	−0.654
Altitude (A)	−0.427	−0.420
Temperature (T)	0.516	0.427
Dissolved oxygen (DO)	0.248	0.373
A×T	−0.239	−0.275
A×DO	−0.464	−0.337
T×DO	−0.540	0.513

**TABLE 8:** Principal component analysis.

Factors	Contribution in U_crit_ (bl/s) (%)	Contribution in U_burst_ (bl/s) (%)
Body length	7.71	8.57
Altitude (A)	15.39	14.77
Temperature (T)	14.72	15.13
Dissolved oxygen (DO)	15.89	15.33
A×T	16.19	16.00
A×DO	14.86	14.85
T×DO	15.24	15.34

## Discussion

For the subfamily Schizothoracinae, these results on swimming ability (Table 1; U_crit_: 2.22–8.48 bl/s, and U_burst_: 3.35–13.20 bl/s) are similar to Hou et al. (2018) who reported a U_crit_ range from 2.07 bl/s to 7.99 bl/s and a U_burst_ range from 2.95 bl/s to 12.46 bl/s for 16 Schizothoracinae species. Based on the intra group results of the three groups of altitude experiments (Figures 1, 2), the swimming ability (U_crit_ and U_burst_, bl/s) decreased with increasing body length. This is consistent with results reported in the literature and reflects the allometry between fish body size and swimming ability (Brett and Glass, 1973; Verhille et al., 2014; Katopodis et al., 2019; Cai et al., 2020).

The spatial distribution of biodiversity is determined by environmental conditions and biological evolution, and the primary environmental factors for fish are temperature, precipitation, and habitat area (Gaston, 2000; Rahbek, 2006). Altitude affects river water temperature and DO level, both of which affect fish physiology. The fish tested in this study were all local Schizothoracinae species caught and tested during their spawning migration. The differences in water temperature and DO associated with altitude difference significantly affected the U_crit_ and U_burst_ of the tested fishes, which is supported by Table 8 indicating that except for body length, all variables have a similar effect on swimming ability.

In this study, water temperature decreased with altitude, as did fish swimming ability. The decrease in swimming ability with decreasing water temperature is consistent with previous research (Ojanguren and Brana, 2000; Penghan et al., 2014). The decrease can be estimated using the swimming ability-water temperature model reported by Ojanguren and Brana (2000). At low temperatures, physiological processes in fish slow down, as indicated by decreased enzyme activity and lower concentrations of adenosine triphosphate, phosphocreatine and glucose (Johnson and Bennett, 1995). These changes, in turn, reduce muscle contraction capacity and decrease fish swimming ability.

The saturation value of DO in water decreases with altitude and low DO levels influence not only oxygen availability, but fish gill morphology and ion regulation (osmotic balance), which then affects fish respiration and metabolism (Matey et al., 2008). Given basic assumptions about metabolism and the cost of transport, our results are again consistent with previous research. Davis et al. (1963) and Penghan et al. (2014) reported that fish swimming ability decreases as DO level decreases. Atmospheric pressure decreases with increasing altitude, resulting in a lower equilibrium concentration of DO in water. However, water temperature also decreases with increasing altitude, which increases oxygen solubility in water. Because the relative decrease in pressure is generally much larger than the relative decrease in temperature (Tromans, 1998), DO tends to decrease as altitude increases. In this study, the combined influences of decreasing atmospheric pressure and air temperature with altitude was accounted for by carrying out the tests at altitude.

Principal component analysis and Pearson correlation analysis were conducted to better understand which altitude parameter (temperature, DO) most affected swimming ability. In Table 7, except for body length, the absolute values of the correlation coefficients between T×DO (temperature×DO) and the two measures of swimming ability are the largest. Furthermore, in Table 8, the A×T (altitude×temperature) had the highest explanatory power for the two measures of swimming ability. Combining the results of the two analyses, we conclude that temperature most affects fish swimming ability. Because water temperature is altitude dependent, the effect of temperature on swimming ability can be attributed to altitude.

Pearson correlation analysis, principal component analysis, and the reported effects of water temperature and DO on swimming ability, show unequivocally that fish swimming ability (U_crit_ and U_burst_) decreases with increasing altitude. However, with U_burst_, differences between the low and medium altitude groups, and between the low and high altitude groups were not significant. These results do not follow the stepwise progression with altitude observed with U_crit_ and this is attributed to the relatively high variability (large SD) within the low and medium altitude groups. The higher variability results from a combination of factors; differences between species, the wider range of test fish body length (0.14–0.29 m in the 1326 m group), and the less representative 20 s time interval in the U_burst_ test *versus* the 15 min time interval in the U_crit_ test. To reduce the risk implied by this uncertainty, the swimming ability of different species and sizes of fish should be considered when designing fishways.

To be effective, a fishway must allow target species to successfully pass, and knowledge of fish swimming capability is crucial for effective fishway design (Peake et al., 1997; Rodgers et al., 2017; Katopodis et al., 2019). However, the effects of high altitudes on fish physiology go beyond the effects produced by lower temperature and DO level. Any chemical reaction or biochemical process that involves gaseous reactants or products is pressure dependent, including crucial processes such as the dissolution of gases in blood and the binding of oxygen by hemoglobin. Although documentation is lacking, we are aware of instances in which data from fish swimming tests conducted at low altitudes were used by engineers to design high-altitude fishways. Therefore, we recommend that fish swimming ability be tested at the same or similar altitude as the target fishway site to ensure the validity of the fish data used for fishway design.

## Data Availability

The original contributions presented in the study are included in the article/supplementary material, further inquiries can be directed to the corresponding author.
